# Indoor Household Exposures and Associated Morbidity and Mortality Outcomes in Children and Adults in South Africa

**DOI:** 10.3390/ijerph19159471

**Published:** 2022-08-02

**Authors:** Oyewale Mayowa Morakinyo, Matlou Ingrid Mokgobu

**Affiliations:** 1 Department of Environmental Health, Faculty of Science, Tshwane University of Technology, Private Bag X680, Pretoria 0001, South Africa; mokgobumi@tut.ac.za; 2 Department of Environmental Health Sciences, Faculty of Public Health, College of Medicine, University of Ibadan, Ibadan 200284, Nigeria

**Keywords:** indoor household exposure, air pollution, children and adults, morbidity and mortality outcomes, South Africa

## Abstract

Human exposure to indoor pollution is one of the most well-established ways that housing affects health. We conducted a review to document evidence on the morbidity and mortality outcomes associated with indoor household exposures in children and adults in South Africa. The authors conducted a scientific review of the publicly available literature up to April 2022 using different search engines (PubMed, ProQuest, Science Direct, Scopus and Google Scholar) to identify the literature that assessed the link between indoor household exposures and morbidity and mortality outcomes in children and adults. A total of 16 studies with 16,920 participants were included. Bioaerosols, allergens, dampness, tobacco smoking, household cooking and heating fuels, particulate matter, gaseous pollutants and indoor spray residue play a significant role in different morbidity outcomes. These health outcomes include dental caries, asthma, tuberculosis, severe airway inflammation, airway blockage, wheeze, rhinitis, bronchial hyperresponsiveness, phlegm on the chest, current rhinoconjunctivitis, hay fever, poor early life immune function, hypertensive disorders of pregnancy, gestational hypertension, and increased incidence of nasopharyngeal bacteria, which may predispose people to lower respiratory tract infections. The findings of this research highlight the need for more initiatives, programs, strategies, and policies to better reduce the negative consequences of indoor household exposures.

## 1. Introduction

Housing is an important factor that influences one’s health and quality of life [1]. Housing is intended to give shelter as well as protection from physical and social environmental threats [2]. The indoor environment has a significant impact on human well-being because most individuals spend 90% of their time indoors, primarily at home [3]. Human exposure to indoor air pollution is one of the most well-established ways that housing affects health [4].

Indoor pollution and its consequences on human health have piqued international interest. Inside houses or buildings, indoor air pollution can arise from occupants’ activities such as cooking, smoking, using electronic machines, or using indoor residual spraying [5], consumer products, or construction materials [5]. The use of solid fuels for cooking and heating, as well as tobacco smoking, are the main contributors to indoor air pollution in low and middle-income nations [6]. Moreover, pollution of indoor air can come from a variety of sources, including biological, chemical, and physical sources [7].

Bioaerosols are a mix of bacteria, fungi, viruses endotoxins and their metabolites and toxins dispersed in the air. They make up roughly 5% to 10% of airborne particulate matter and are ubiquitous in the environment [7,8]. Indoor bioaerosols can cause allergies and be harmful to one’s health [8]. Mucous membrane irritation, weariness, headaches, memory loss, and infant bronchiolitis can all be caused by a large number of fungi and bacteria in the indoor environment [9].

Many individuals smoke throughout the world despite the weight of research pointing to the detrimental consequences of tobacco use on health [10]. Moreover, everyone’s health is negatively impacted by exposure to secondhand smoke (SHS), and there is no known “safe” level of exposure [11]. Around the world, SHS-related illnesses claim roughly 1.2 million nonsmokers’ lives each year [12]. Developing nations, particularly those in Africa and Asia, account for two-thirds of these deaths [12]. Exposure to SHS has been associated with lung cancer [13], breast cancer [14], stroke [15] and other cardiovascular diseases [16]. SHS exposure in children results in bronchitis, pneumonia, asthma, otitis media (ear inflammation), and sudden infant death syndrome [17].

Almost 2.6 billion people worldwide, according to the World Health Organization (WHO), rely on polluting fuels like wood, coal, crop waste, animal dung, or charcoal for cooking and heating, which are combined with inefficient stoves [18]. Approximately 83 percent of the population in the WHO African area is estimated to rely mostly on polluting cooking methods [19]. Wood fuels are the most commonly used solid fuels in sub-Saharan Africa (SSA). The number of families who still use traditional sources of solid fuels for heating and/or cooking is highest in SSA [20]. These fuels burn inefficiently, resulting in significant levels of gaseous and particle pollution in homes [21]. They produce significant amounts of household air pollution, including microscopic soot particles that can penetrate deep into the lungs [21].

**Figure 1:**
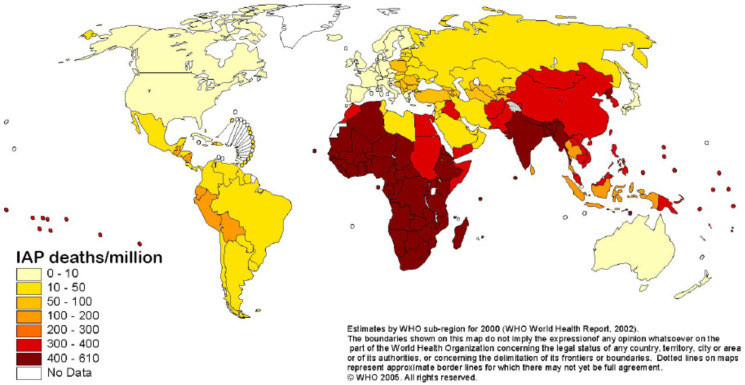
Distribution of deaths from indoor smoke from solid fuels. Source: WHO [37]. Note: IAP means Indoor air pollution.

Indoor air pollution from solid fuel usage is the single most important environmental risk factor for disease worldwide [22,23], and one of the top ten causes of death [24], particularly among the poorest people in low- and middle-income nations [25]. Nearly 4 million people die prematurely each year as a result of household air pollution caused by poor cooking habits with polluting stoves fuelled by solid fuels and kerosene (Figure 1) [18]. Household air pollution nearly doubles the risk of pediatric pneumonia, accounting for 45 percent of all pneumonia deaths in children under the age of five. Adults are at risk for acute lower respiratory infections (pneumonia) from household air pollution, which accounts for 28% of all pneumonia deaths [18].

South Africa is an upper-middle-income country with a population of 60,848,809 in 2022 based on the most recent United Nations estimates [26]. A countrywide study conducted in 2017 found that about 20% of South Africa’s adult population (>15 years of age or older) were smokers [27]. Over time, more men than women smoke in South Africa [28,29,30]. Approximately 9% of deaths in the nation were associated with smoking [31]. The expense of tobacco consumption ranges from roughly 31 to 60 billion Rand per year [32,33].

Moreover, in South Africa, the domestic burning of coal, wood, and paraffin (also known as kerosene) for cooking and heating is the main source of indoor air pollution [34]. Indoor air pollution is a major issue in rural areas of South Africa, where clean energy sources are scarce [35]. Furthermore, low-cost coal can be found near coal mines and coal-fired power plants in the country’s interior [34]. In these communities, coal is typically used for heating and cooking, with electricity being utilized for illumination. Because wood and coal are scarce in some coastal areas along South Africa’s eastern coast, paraffin is commonly used for heating and cooking [36].

The reduction of the burden of illnesses and deaths caused by air pollution has been identified as a key agenda for sustainable development and has been designated as Sustainable Development Goal 3.9 [38]. Because indoor air pollution can come from a variety of sources, including biological, chemical, and physical sources, as well as biomass and environmental tobacco smoking, evidence of the link between indoor household exposures and associated morbidity and mortality outcomes are needed. The findings of this research will highlight the need for more initiatives, programs, strategies, and policies to better reduce the negative consequences of indoor household exposures. We conducted a review to document evidence on the morbidity and mortality outcomes associated with indoor household exposures in South Africa.

## 2. Materials and Methods

### 2.1. Search Strategy

The authors conducted a scientific review of the publicly available literature up to April 2022. We used the advanced search option of different search engines (PubMed, ProQuest, Science Direct, Scopus and Google Scholar) to identify the literature that assessed the link between indoor household exposures and morbidity and mortality outcomes in children and adults in South Africa.

### 2.2. Inclusion and Exclusion Criteria

Epidemiological studies in humans living in South Africa that measured exposure to pollutants in biomass (coal, wood, and paraffin), environmental tobacco smoke, bioaerosols and allergens, and indoor spray residue and investigated health outcomes as a risk factor for any observed health effects were included. Case-control, retrospective cohort, surveys, and/or cross-sectional studies were among the studies that were considered. Children and adult studies of either gender were included. Case studies, case reports, editorials, reviews, and commentaries were all excluded from the study. Papers that reported links between indoor home exposure and any health outcomes in the South African population were included in the health outcomes category.

### 2.3. Study Selection

Two reviewers separately checked the titles and abstracts of articles in line with the inclusion and exclusion criteria. A full-text review was performed on those identified as potentially relevant or unclear. Any differences between reviewers about which papers were eligible for inclusion in the review were resolved by discussion or consultation with another member of the research team.

### 2.4. Data Extraction

Data were retrieved from selected articles using a standardized data extraction form. Author, publication year, study design, study population, sample size, study location, exposure assessment, outcome and outcome assessment method, and morbidity and mortality outcomes were among the data retrieved.

## 3. Results and Discussion

### 3.1. Study Characteristics

**Figure 2:**
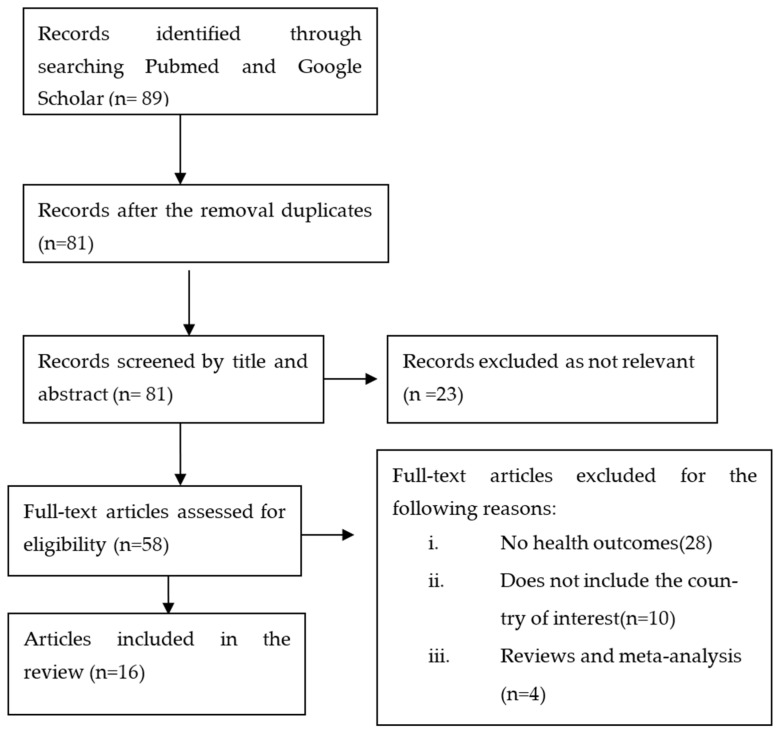
Flow chart of the literature search and selection process.

After deleting duplicates, a total of 89 studies were screened for titles and abstracts. Based on eligibility criteria, full texts from 58 studies were retrieved and examined. Finally, 16 studies with 16,920 participants were included. Figure 2 shows the entire list of studies that were included, while Table 1 shows the study characteristics in detail.

### 3.2. Bioaerosols, Allergens Exposure and Health Outcomes

Biomass fuels and combustion pollutants have been studied in developing countries’ residential indoor settings. In Africa, biological contaminants including allergens have received little attention [42]. Indoor air bioaerosols and allergen exposure has been linked to asthma and other respiratory illnesses [8,9]. Exacerbations and episodes of asthma are most common at home, suggesting that the indoor home environment is a significant factor in asthma outcomes [48,53,54]. The prevalence of pediatric asthma continues to rise in developed and developing countries. Africa is currently ranked 25th in the world in terms of asthma prevalence, with the fifth greatest number of asthma-related deaths among children [48].

In a study conducted in Durban, KwaZulu-Natal, South Africa, by Jafta et al. [42], concentrations of airborne fungus and allergens were measured in the houses of primary school pupils. The association between household variables and pollutant concentrations was also investigated, with the goal of better understanding the link between allergens and pediatric asthma [42]. *Cladosporium* concentrations were highest indoors at 3500 colony forming units per cubic meter of air (CFU/m^3^) and outdoors at 4040 CFU/m^3^; however, these values are underestimates due to sampling overload. *Cladosporium* concentrations exceeding 1000 CFU/m^3^ were found in 30% of the homes, a concentration that has been linked to poor respiratory health effects [55]. In the sleeping areas, *Cladosporium*, *Penicillium* and *Aspergillus* detected can penetrate the bronchi and trigger allergic responses in the lower respiratory tract [56,57]. 

In the same study conducted by Jafta et al. [42], the fungal allergen Asp f1 was detected in all of the dwellings sampled (126 homes), whereas cockroach allergen Bla g1 was present in half of the homes. In 3% and 13% of the sampled homes, respectively, house dust allergens Der f1 and Der p1 exceeded concentrations linked to sensitization and asthma exacerbation risk, whereas Bla g1 exceeded recommendation values in 13% of the homes. Exposure to elevated concentrations of fungus allergen (Asp f1, Alt a1, Cla h1, *S. chartarum*), house dust mite allergen (Der p1 and Der f1), cockroach allergen (Bla g1, Bla g2, Per f1) and animal allergen (Mus m1, Rat n1, Can f1, Fel d1) are linked to asthma aggravation in children (Asp f1, Alt a1, Cla h1, *S. chartarum*) [58,59]. Airborne fungal growth was predicted by moisture, ventilation, floor type, and bedding type. Durban’s annual average temperature (23 °C) and humidity (75%) levels are high, and it is suitable for the growth and multiplication of allergen-producing organisms [42]. This may explain why asthma prevalence rates were observed to be high (32%) among children attending schools in south Durban, compared to a prevalence rate of 17% among children residing outside the area [60].

Another study conducted in the Western Cape province of South Africa to investigate the association between asthma and common indoor exposures among schoolchildren shows that clinically severe airway inflammation (FeNO > 35 ppb) and airway blockage (FEV_1_ < LLN) were seen in a somewhat larger proportion of patients—10.2 percent and 17.6 percent, respectively [48]. The level of dampness in the residence was linked to a twofold increase in the likelihood of having a present wheeze (aOR = 2.60, 95% CI: 1.18–5.71). Rhinitis and household dampness (aOR = 3.00 95% CI: 1.47–6.13) showed a similar pattern, as did mold growth (aOR = 3.37 95% CI: 1.69–6.71). These findings show that rhinitis and wheezing are more common in pupils from low-income informal settlements who live in moist houses with mold growths. Western Cape’s weather and environment are characterized by cold, wet winters and hot, dry summers, which can exacerbate asthma symptoms in children and the elderly [48].

### 3.3. Household/Tobacco Smoking and Dental Caries

Due to its global distribution and severe consequences, dental caries remains one of the most common oral disorders, impacting one-third of the world’s population [61,62]. Dental caries is a complex, dynamic disease caused by biofilms and sugar that causes phasic demineralization and remineralization of dental hard tissues. A significant association between smoking or secondhand smoke exposure and dental caries has been reported in the literature [63,64,65,66].

In South Africa, Ayo-Yusuf et al. [41] reported that an association exists between household members’ smoking or secondhand smoke exposure and caries. Caries in the second molars were more common in participants from smoking homes and significantly linked to caries in permanent teeth of adolescents, independent of sugar intake. These findings are consistent with the findings of other studies that reported an association between secondhand smoke exposure and caries in the permanent teeth literature [63,64,65,66]. For instance, Goto et al. [64] reported that more than three pack-years of mother’s smoking (OR = 5.55, 95% CI: 2.17–14.22, *p* < 0.001) and more than five pack-years of smoking by all family members (OR = 2.00, 95% CI: 1.12–3.58, *p* = 0.004) were substantially related with dental caries. Nicotine also promotes extracellular polysaccharides which might attract other microbes to the tooth plaque such as *Candida albicans* [64].

Several explanations support the biological plausibility of the link and possibly explain how passive cigarette exposure causes caries. Tobacco smoking has a direct impact on both the mineralization of developing teeth and the microbes that live on them [67,68]. Tobacco use is known to be associated with higher levels of *Streptococcus mutans* and *Lactobacillus acidophilus* [69,70]. The immune system’s impairment could make it easier for *Streptococcus mutans* to colonize and lower vitamin C levels in children who have been exposed [71]. Nicotine, present in tobacco, promotes biofilm development and metabolic activity in *Streptococcus mutans* biofilms [71]. Nicotine also promotes extracellular polysaccharides, which might attract other microbes to the tooth plaque, such as *Candida albicans* [72].

Children exposed to tobacco use had lower salivary pH, buffer capacity and saliva flow than non-exposed children, leading to a reduction in the capacity of saliva to protect against caries; this, together with a rise in *S. mutans* and *Lactobacilli* levels, could also explain the cause and effect relationship between tobacco and caries [69].

### 3.4. Household/Tobacco Smoking and Respiratory Outcomes

While smoking is declining in some regions of the world [73], it is increasing in others, notably in low and medium-income countries (LMICs). Public smoking restrictions may not prevent smoking in households, where women and children may be exposed to secondhand smoke (SES) from household members [74,75,76]. Second-hand smoke is the smoke that lingers in the air after a smoker has exhaled (SHS). The death toll from exposure to secondhand smoke is over 1.2 million per year [10]. In comparison to only approximately 12% of women globally, more than 40% of men consume tobacco [77]. According to this global profile, the majority of those exposed to SHS are women and children. Due to these differences in smoking rates between the sexes and the fact that women and children spend more time at home than men, as well as other prevailing conditions in their homes, women and children in developing nations are more likely to be exposed to secondhand smoke [78]. A study conducted in 31 countries on secondhand smoke exposure among women and children revealed that mothers and children exposed to SHS have a higher risk of early death and disease than those who do not [79].

In the Matlosana district townships surrounding Klerksdorp, South Africa, a high prevalence of air pollution from secondhand tobacco smoke was recorded among individuals in homes with a case of prevalent active tuberculosis (TB) disease [45]. Adults in 40.0% of homes reported a daily smoker in the home, and 70% of homes had detectable air nicotine. SHS was found in 83.0% of houses with a history of TB compared to 65.0 percent of homes without a TB history (65.0%). SHS may contribute to the spread of TB.

Moreover, Olaniyan et al. [48] reported asthma-related health outcomes among school children exposed to indoor air pollutants in two municipalities in the Western Cape province of South Africa. In adjusted logistic regression models, passive smoking was associated with a two- to three-fold increased risk in upper and lower airway outcomes. Having a smoker in the home significantly increased the odds of current wheezing (aOR: 1.79, 95% CI: 1.02–3.15).

In a related study by Ehrlich et al. [39], a high prevalence (47.0%) of undiagnosed and untreated childhood asthma was reported in Cape Town. Asthma exacerbation and episodes occur mostly at home, especially during the weekends, indicating the indoor home environment to be an important contributor to asthma-related outcomes [80]. Africa currently ranks 25th globally in asthma prevalence and has the fifth-highest number of deaths due to asthma among children [81]. Vanker et al. [47] also reported a significant association between exposure to particulate matter and lower respiratory tract infection (LRTI) (OR = 1.43, 95% CI: 1.06–1.95; *p* = 0.008). In a cohort study by Vanker et al. [47], wheezing in children was significantly associated with maternal passive smoke exposure (1.70, 1.25–2.31; *p* = 0.001) and with any household member smoking (1.55, 1.17–2.06; *p* = 0.002) in South Africa.

Ehrlich et al. [39] also reported an adverse effect of maternal smoking on lung function in asthmatic children in Cape Town, South Africa. Forced Expiratory Volume 1 (FEV1) was lower among children whose mothers currently smoked. However, in contrast to other studies, children with asthma whose mothers smoked had a lower frequency of bronchial hyperresponsiveness (BHR) than asthmatic children of nonsmoking mothers, particularly if the mother smoked ≥15 cigarettes daily. BHR was also less common among children sharing a house with four or more smokers vs. fewer or none. BHR was unrelated to paternal smoking [39].

Furthermore, Shirinde and colleagues conducted a study to determine the association between wheezing and selected air pollution sources in Ekurhuleni Metropolitan Municipality, South Africa. The findings revealed that children who were exposed to environmental tobacco smoke (ETS) in Tembisa were 14.0% more likely to develop current wheeze (OR = 1.36, 95% CI: 1.06–1.77) than those not exposed to ETS [43].

In a study conducted in South Africa on determining the effects of indoor air pollution and tobacco smoke exposure on nasopharyngeal bacterial carriage in mothers and infants, antenatal ETS exposure was associated with *Streptococcus pneumoniae* carriage in mothers (adjusted risk ratio (aRR = 1.73, 95% CI: 1.03–2.92)) while postnatal ETS exposure was associated with carriage in infants (aRR = 1.14, 95% CI: 1.00–1.30). In infants, ETS exposure was also associated with an increased risk of *S. pneumoniae* carriage at 6 months of age (aRR = 1.14, 95% CI: 1.00–1.30). The association between smoke exposure and *S. pneumoniae* was also noted when adjusting for the other bacterial organisms co-carried (aRR = 1.16, 95% CI: 1.02–1.32) at 6 months [52].

The link between nasopharyngeal carriage of *S. pneumoniae* and subsequent *S. pneumoniae* illness, particularly LRTIs, is well known [82,83,84]. Environmental tobacco smoke exposure may influence nasopharyngeal carriage of bacterial species and the development of LRTI [52]. In mice models, cigarette smoke inhibited the expression of nasal inflammatory mediators which are normally activated by *S. pneumoniae* carriage, predisposing to invasive *S. pneumoniae* infection. Tobacco smoke contains a wide range of chemicals and carcinogens, all of which have the potential to harm a child’s growing respiratory system. For instance, nicotine (present in tobacco) is known to have a severe negative impact on lung development and collagen deposition [85]. During the pseudoglandular phase, nicotine stimulates alpha-7 nicotinic acetylcholine receptors, causing dysanaptic lung growth [86]. Furthermore, there is evidence associating tobacco smoke exposure with poor early life immune function, causing an imbalance in Th1 and Th2 responses, thus increasing the risk of allergy disorders and children’s respiratory infections [85,87].

Ngobese et al. [88] have reported in their study on non-smokers’ exposure to second-hand smoke in South Africa that nonsmokers’ exposure to tobacco smoke is significant (47.0%) in South Africa. The majority of participants had been exposed to SHS at home, and these individuals are the youngest of the age groups. These results are concerning because research has indicated that children or young people who are exposed to SHS at home are more likely to start smoking later on as a result of the normalization of smoking by their close relatives [89].

Nonetheless, in the year 2005, South Africa joined the WHO Framework Convention on Tobacco Control (FCTC) and is therefore legally required to put in place and uphold regulations that shield nonsmokers from unintentional exposure to SHS [90]. Furthermore, the South African Tobacco Products Act, 83 of 1993 (amended in 2008), stated that no person is to smoke in a private dwelling that is used for commercial childcare activity (e.g., schooling and tutoring) [91]. Thus, residents are allowed to smoke within their homes, barring any spaces used for childcare. The implication of this is that there are not enough initiatives to promote smoke-free homes and raise awareness about the dangers of SHS exposure. There is a critical need for South Africa to increase public awareness of the dangers of SHS and enact rules that would discourage smoking in indoor home environments [88].

### 3.5. Household Cooking and Heating Fuels and Adverse Health Outcomes

The most common and significant cause of air pollution is the combustion of non-electric fuels (wood, charcoal, dung, agricultural residues, and other raw plant material) for cooking, heating, or both [43]. When biomass fuels (non-electric fuels) are burned in basic open stoves or fires with incomplete combustion, significant pollutants such as carbon monoxide, particulate matter, and volatile organic compounds (e.g., benzene, benzo(a)pyrene) are released [92,93]. In homes that rely on biomass fuel for cooking and heating, poor ventilation may also contribute to high levels of indoor pollution [94].

In a study conducted in South Africa to investigate whether the use of cooking and heating fuel significantly increases the risk of dying during the first 1–59 months of life, children in households using polluting fuels had a significantly higher risk of dying: from 2.22 (95% CI = 1.22–4.04; *p* = 0.009) to 1.95 (95% CI = 1.04–3.68; *p* = 0.039) in the univariate and adjusted analyses, respectively. The overall mortality incidence rate in the five years preceding the study was 1.473 per 1000 person months. Two-thirds of the children lived in rural households where 75% of the deaths occurred. Seventy-nine percent of these children were from households that used polluting fuels for cooking and heating, either alone or in combination with clean fuels [40].

In a study conducted by Albers et al. [44], in two towns in Mpumalanga in South Africa, an association between household fuel use and childhood respiratory morbidity. A higher prevalence of respiratory outcomes was reported among users using non-electrical fuels for cooking than users of electricity [44]. The prevalence of phlegm in the chest was higher among coal users (36.9%) than electricity users (23.5%). Furthermore, in homes where wood and coal were predominantly used, a higher prevalence of phlegm on the chest (29.3%) and bronchitis (19.7%) was recorded than 15.8% (phlegm on the chest) and 11.1% (bronchitis) among users of electricity. Increased prevalence of chest cough (25.7%) was evident among users of paraffin for cooking. The prevalence of wheezing (20.9%) and asthma (11.6%) among paraffin users was higher than the prevalence of wheezing (16.0%) and asthma (7.1%) among electricity users [44].

In another study carried out in Mpumalanga, South Africa, Bidassey-Manilal et al. [50] reported that wood was the most commonly used fuel for cooking (75.0%) and heating (69.0%). Coal is the second most commonly used fuel for cooking (15.0%) and heating (14.0%). The prevalence of rhinitis ever, current rhinitis, and current rhinoconjunctivitis was 67%, 70%, and 69%, respectively. The odds of rhinitis ever (OR = 1.21, 95% CI: 1.05–1.46), current rhinitis (OR = 1.26, 95% CI: 1.01–1.40), and hay fever (OR = 1.11 95% CI: 1.21–1.48) were higher among children living in households that are users of wood, coal and kerosene. The probability of developing rhinitis (OR 1.31, 95% CI: 1.04–1.69) and hay fever (OR 1.21, 95% CI: 1.07–1.81) increases with the use of wood, coal and kerosene for cooking in the presence of children [50]. However, there was no association between indoor smoking, the use of animal dung, coal, charcoal and electricity and respiratory health outcomes.

In four informal settlements in the Western Cape province in South Africa, the use of paraffin for cooking or heating was significantly associated with asthma occurrence [48]. There was a twofold likelihood of occurrence of significant airway inflammation (aOR: 2.31, 95% CI: 1.05–5.06) and an increased risk of rhinitis (aOR: 1.69, 95% CI: 1.05–2.70) from exposure to paraffin among schoolchildren. This finding is consistent with the findings of another study in Polokwane, South Africa that reported an increased risk of asthma (OR: 1.50, 95% CI: 1.09–2.10) among school children living in homes using predominantly biomass fuel for cooking [95].

Among rural South African women using wood for cooking, higher odds (OR = 1.41; 95% CI = 0.72–2.77) of self-reported wheezing/chest tightness were reported compared with electricity users [49]. There was no indication of a link between wood-fired cooking and self-reported dyspnea, respiratory diseases, or pre-hypertension/hypertension. Minimal evidence of an effect of cooking with wood on blood pressure (systolic β = −0.33, 95% CI = −2.37, 1.71; diastolic β = −0.21, 95% CI = −1.77, 1.35) was found [49].

A related study conducted by Shirinde and others [43] on determining the association between air pollution sources and the occurrence of wheeze revealed that Gas was the most frequently used fuel for heating and was significantly associated with wheeze ever (OR 1.47, 95% CI: 1.15–1.88). In Tembisa, gas was mostly used for heating homes and was associated with wheeze ever (OR 1.68, 95% CI: 1.23–2.28) and current wheeze (OR 1.61, 95% CI: 1.08–2.39). However, in Kempton Park, gas was mostly used for residential cooking and it was also associated with wheeze ever (OR 1.65, 95% CI: 1.04–2.61). An association between paraffin use and current severe wheeze (OR 1.85, 95% CI: 1.04–3.28) was observed in households where paraffin is most commonly used for residential heating [39].

In Umlazi, a low-income and informal settlement in South Africa, the use of non-electric fuels (coal, wood, gas, paraffin) and electricity are the major sources of energy for cooking and heating [34]. There are significant odds of having upper respiratory tract infections (URTIs) from exposure to non-electric sources for cooking (aOR = 2.9, 95% CI: 1.1–7.9, *p* < 0.05) and heating (aOR = 3.6, 95% CI: 1.2–10.1, *p* < 0.05). Furthermore, the prevalence of lower respiratory tract infections (LRTIs) was significantly associated with electric energy use for heating (aOR = 2.7, 95% CI: 1.1–6.4, *p* < 0.05).

The use of solid fuels and kerosene may play an important role in perpetuating the tuberculosis (TB) epidemic. In their study conducted in Klerksdorp, South Africa, Elf et al. [45] recorded a high prevalence of air pollution from solid fuels and kerosene use among persons in homes with a case of prevalent active TB disease. Solid/kerosene fuel use for more than 1 h/day was more prevalent in homes with a history of previous TB (27%) than those without previous TB (21%). Nearly one-third of households reported any burning of wood or kerosene for cooking or heating.

Inflammation and oxidative stress are thought to be the mechanisms by which biomass-related household air pollution harms people’s health [94]. In vitro [96] and experimental [97,98,99] studies have shown that short-term controlled inhalation exposure to wood smoke can cause pulmonary and systemic inflammation.

### 3.6. Particulate Matter, Gaseous Pollutants and Adverse Health Outcomes

Unclean fuels occupy the bottom of the energy ladder in terms of combustion efficiency and cleanliness, and burning them releases health-harming chemicals (PM_10_, PM_2.5_, NO, NO_2_, SO_2_, CO, benzene, formaldehyde, polycyclic aromatic hydrocarbon among others) into the atmosphere.

Particulate matter (PM) can range in size from a few nanometers (nm) to tens of micrometers (μm) [100]. PM_0.1_ (ultrafine fine particles of an aerodynamic diameter of less than 0.1) have a large surface area and can penetrate deep into the lungs, PM_2.5_ (fine or respirable particles of aerodynamic diameter of less than 2.5) can penetrate to the gas exchange region of the lung while PM_10_ (coarse or inhalable particles of aerodynamic diameter of less than 10) can penetrate into the human respiratory system [101]. Even though there is minimal evidence that shows a threshold below which no adverse health effects would be expected, the WHO reported that negative health impacts of PM_2.5_ are prevalent in the respiratory and cardiovascular systems [102]. The lowest concentration range that has been shown to have harmful health consequences is predicted to be 3 to 5 gm^3^ [102]. According to epidemiological studies, PM_2.5_ is the primary cause of respiratory health consequences in humans [103,104].

In Durban, South Africa, Gumede and Savage [46] reported that indoor PM_2.5_ concentration levels were found to have a high relationship (*p* < 0.002) with percent anticipated forced vital capacity (FVC). The spirometry test revealed that the majority of the children who took part in the test had poor lung function.

Moreover, exposure to indoor air pollution or environmental tobacco smoke may affect bacterial transport in the nasopharynx and the development of lower respiratory tract infections. In a study conducted in South Africa by Vanker et al. [52], increased maternal nasopharyngeal carriage of *M. catarrhalis* was linked to antenatal exposure to NO_2_ levels above ambient levels when adjusted for all clinical confounders (aRR = 3.65, 95% CI: 1.39–9.58) and additional pollutants (aRR = 3.65, 95% CI: 1.39–9.58). Exposure to benzene is associated with the occurrence of maternal *H. influenzae* carriage [38]. Furthermore, exposure to cigarette smoke nearly quadrupled the chance of *S. pneumoniae* carriage (aRR = 1.73, 95% CI: 1.03–2.92). In newborns, postnatal particulate matter exposure was linked to nasopharyngeal carriage of *H. influenza* (aRR = 1.68, 95% CI: 1.10–2.57) or *Moraxella catarrhalis* (aRR 1.42, 95% CI: 1.03–1.97). Environmental exposures during infancy have been linked to an increased incidence of particular nasopharyngeal bacteria, which may predispose people to LRTI [83,84,105].

The findings of this study that an association exists between PM_10_ exposure and *H. influenzae* and *M. catarrhalis* carriage in infants were consistent with other studies [105,106]. PM_10_ exposure is well known to be linked to LRTIs and pediatric respiratory illnesses. By disrupting human defensins, a key component of antimicrobial action, PM_10_ aided bacterial invasion of epithelial airway cells by weakening innate defense systems [74].

### 3.7. Indoor Spray Residue and Adverse Health Outcomes

The application of insecticides inside houses for malaria prevention, known as indoor residual spraying, may result in increased exposure to pesticides such as dichlorodiphenyltrichloroethane (DDT). In a study conducted in South Africa at the time of birth, 733 rural South African women who took part in the Venda Health Examination of Mothers, Babies, and Their Environment were tested for DDT and its breakdown product dichlorodiphenyltrichloroethylene (DDE) [5]. Based on self-reports and data extracted from medical records, there was an association between maternal DDT/E serum concentrations and an increased risk of hypertensive disorders of pregnancy (HDP) diagnosis. Similarly, based on medical records, p,p′-DDE was most strongly linked with HDP (OR = 1.47; 95 percent CI = 1.03, 2.09) and gestational hypertension (OR = 1.44; 95% CI: 1.00–2.07) diagnosis, whereas p,p′-DDT was most strongly associated with HDP (OR = 1.32; 95% CI: 0.99–1.75).

Mechanistic data support the possibility that DDT has a causal influence on HDP and hypertension. DDT, for example, activates the renin-angiotensin system [107], a signaling pathway that may have a role in hypertension by mediating the formation of reactive oxygen species [108]. DDT and DDE have also been linked to preeclampsia because of their ability to promote oxidative stress and endothelial cell dysfunction [109,110]. DDT and DDE are lipid soluble and can pass the human placenta, making them very persistent in biological tissues and the environment [111].

### 3.8. Household Dampness and Respiratory Effects

In a study conducted in Durban, South Africa by Jefta et al. [51], household dampness was significantly associated with childhood pulmonary tuberculosis (PTB) in unadjusted (OR = 1.8, 95% CI: 1.01–3.1) and adjusted (aOR = 2.4, 95% CI: 1.1–5.0) analysis. Although dampness has been linked to fungal respiratory infections [112,113], no studies have linked it to pulmonary tuberculosis (PTB). Dampness has been linked with mold growth in the indoor environment [114]. Mold has been found to exacerbate respiratory discomfort or colonize cavities in the lungs caused by tuberculosis [115]. As a result, exposure to mold or moisture exposes the respiratory system to mycotoxins, glucans, and volatile organic compounds, all of which weaken immunity. The mycobacterium in the lungs is controlled by mycotoxin, which affects defensive mechanisms [115].

## 4. Conclusions

Our review examined the literature on health morbidity and mortality outcomes associated with indoor household exposure in South Africa. Though both rural and urban areas are becoming more electrified, many South African homes still rely on alternative energy sources such as wood fuel, gas, coal, and paraffin. People who have access to electricity, on the other hand, use biomass fuels to augment their energy consumption, such as using electricity for lighting but wood and coal for cooking and heating, to keep their electricity bills down.

Our study highlights that disease burden is influenced by bioaerosols, allergens, dampness, cigarette smoking, domestic cooking and heating fuels, particulate matter, gaseous pollutants, and indoor spray residue. The outcomes associated with indoor household exposure include dental caries, asthma, tuberculosis, severe airway inflammation, airway blockage, wheeze, rhinitis, bronchial hyperresponsiveness, phlegm in the chest, current rhinoconjunctivitis, hay fever, poor early life immune function, hypertensive disorders of pregnancy, gestational hypertension, and increased incidence of nasopharyngeal bacteria, which may predispose to lower respiratory tract infections.

Our research emphasizes the critical importance of evidence-based policymaking and decision-making to reduce the significant burden of disease associated with home air pollution, especially among children and adults.

Some drawbacks must be noted in the interpretation of the results. In most of the studies reviewed, the use of a questionnaire for exposure assessment was paramount. This exposure assessment technique does not provide accurate estimates of individual exposure. Furthermore, most of the studies used cross-sectional designs, making it difficult to determine a causal and time-related association between exposure to indoor household exposures and health outcomes. The accuracy of the respondent’s memory for prior information also has a role in the quality of survey results. Inaccuracies in measurement and missing data in some of the variables could result from failure to accurately recollect prior knowledge. However, the generally consistent findings across the various study designs suggest that indoor household exposure is detrimental to the health of South Africans.

## Figures and Tables

**Table 1:** Summary of studies on indoor household exposures and associated morbidity and mortality outcomes in children and adults in South Africa.

SN	Author, Year	Study Design	Study Population	Sample Size	Study Location	Exposure	Morbidity or Mortality Outcome
1.	Ehrlich et al. [39]	Cross-sectional	Children of ages 7–11 years with reported asthma or multiple asthma symptoms	249 children	Cape Town, South Africa	Household environmental tobacco smoke	Reduced lung function
2.	Wichmann and Voyi [40]	Cross-sectional	Under-five children	3556 under-five children living in 2828 households	Nine provinces in South Africa	Exposure to cooking and space heating smoke	Under-five mortality. Children in households using polluting fuels are 2.22 times (95% CI = 1.22–4.04; *p* = 0.009) at risk of dying than those using clean fuels (OR = 1.95, 95% CI = 1.04–3.68; *p* = 0.039)
3.	Ayo-Yusuf et al. [41]	Cross-sectional	High school students	1873 8th-graders	21 randomly selected schools in the most rural of the nine provinces in South Africa	Household tobacco smoke	Dental caries. Secondhand smoke exposure is linked to caries in permanent teeth in teenagers, regardless of sugar consumption.
4.	Jafta et al. [42]	Case-control design	School children (grades 3–6) with known or probable persistent asthma	81 children	Durban, South Africa	Allergens—dust mite (Der p1 and Der f1), fungi allergens (Asp f1) and cockroach allergen (Bla g1)	Asthma
5.	Shirinde et al. [43]	Cross-sectional	Children between the ages of 13 and 14 years	3468	Ekurhuleni Metropolitan Municipality, namely Tembisa and Kempton Park, South Africa	Environmental Tobacco Smoke, Gas and Paraffin for residential heating	Wheeze
6.	Albers et al. [44]	Cross-sectional	Children between the ages of 9 and 11 years	627 children	Mpumalanga Province, South Africa	Fuel used for cooking (electricity, gas, paraffin, wood, charcoal)	Respiratory health outcomes: phlegm on the chest, chest cough, bronchitis, wheezing and asthma
7.	Elf et al. [45]	Cross-sectional	All adults (≥18 years of age) and children between seven and 17 years of age living in the same household as the index Tuberculosis case, including the index case themselves	96 adults and 28 children in 53 households were included	Matlosana district townships surrounding Klerksdorp, South Africa	Secondhand tobacco smoke, use of solid fuels, and kerosene	Tuberculosis
8.	Gumede and Savage [46]	Cross-sectional	Children aged 6 to 12 years	23 children	Clare Estate community in Durban, South Africa	PM_2.5_	Lung function A significant association was observed between the percent predicted forced vital capacity (FVC) and indoor PM_2.5_ concentration levels (*p* < 0.002). Impaired lung function was recorded among children.
9.	Vanker et al. [47]	Cohort study	Mother and infant pairs	1137 mothers with 1143 livebirths	Paarl, South Africa	Particulate matter, nitrogen dioxide, sulphur dioxide, carbon monoxide, and volatile organic compounds benzene and toluene	Exposure to particulate matter was significantly associated with LRTI (OR = 1.43, 95% CI: 1.06–1.95; *p* = 0.008). wheezing was associated with maternal passive smoke exposure (1.70, 1.25–2.31; *p* = 0.001) and with any household member smoking (1.55, 1.17–2.06; *p* = 0.002).
10.	Olaniyan et al. [48]	Cross-sectional	Children between the ages of 9 and 11 years	590 children	Khayelitsha, Marconi-Beam, Masiphumulele and Oudtshoorn in the Western Cape Province of South Africa	Dampness, presence of visible mold growth, pets in the home, smokers in the home, and the use of paraffin for cooking and heating.	Rhinitis, doctor-diagnosed asthma, ocular-nasal symptoms, wheezing and other respiratory symptoms. Paraffin use was associated with a twofold increased likelihood of having significant airway inflammation (aOR: = 2.31, 95% CI: 1.05–5.06) and an increased risk of rhinitis (aOR = 1.69, 95% CI: 1.05–2.70). Having a smoker in the home significantly increased the odds of current wheeze (aOR = 1.79, 95% CI: 1.02–3.15) Dampness in the home was associated with a twofold increased odds of current wheeze (aOR = 2.60, 95% CI: 1.18–5.71). An association was observed between rhinitis and household dampness (aOR = 3.00 95% CI: 1.47–6.13) and visible mold growth (aOR = 3.37, 95% CI: 1.69–6.71).
11.	Misra et al. [49]	Cross-sectional	reproductive-aged women 20–30 years	415 women	Women from eight villages in the Thulamela Municipality of the Vhembe district in the Limpopo Province of South Africa.	Cooking fuel (wood and electricity)	Biomarkers of inflammation, respiratory symptoms (breathlessness, wheezing/chest tightness) and illnesses (tuberculosis, pneumonia, and asthma), and blood pressure. Increased odds (aOR = 1.41; 95% CI = 0.72–2.77) of self-reported wheezing/chest tightness among women who cook with wood, An increased odds of both breathlessness (aOR = 1.29, 95% CI = 0.65, 2.56, *p* > 0.05) and pre-hypertension/hypertension (aOR = 1.29, 95% CI: 0.80, 2.09) among women who reported cooking with wood mostly indoors Wood for cooking has effect on blood pressure (systolic β = −0.33, 95% CI: −2.37, 1.71; diastolic β = −0.21, 95% CI = −1.77, 1.35)
12.	Murray et al. [5]	Cohort	Women participating in the Venda Health Examination of Mothers, Babies and their Environment (VHEMBE) study	733 women	Rural Vhembe District of Limpopo Province, South Africa	dichlorodiphenyl trichloroethane (DDT), dichlorodiphenyl dichloroethylene (DDE)	Hypertension, preeclampsia, or eclampsia. DDT was associated with Hypetensive disorder of pregnancy (HDP) based on self-report (OR = 1.50, 95% CI = 1.10–2.03) and medical records (OR = 1.32, 95% CI = 0.99–1.75), respectively. DDE was associated with HDP based on self-report (OR = 1.58, 95% CI = 1.09–2.28) and medical records (OR = 1.47, 95% CI = 1.03, 2.09), respectively. DDE was also associated with gestational hypertension (OR = 1.44, 95% CI = 1.00–2.07).
13.	Bidassey-Manilal et al. [50]	Cross-sectional	Adult above 18 years	167 households	Mpumalanga Province, South Africa	Coal, wood, kerosene, charcoal animal dung	Allergic rhinitis Children living in households that primarily utilized wood, coal, and kerosene were at risk of developing rhinitis ever (OR = 1.21, 95% CI: 1.05–1.46), current rhinitis (OR = 1.26, 95% CI: 1.01–1.40), and hay fever (OR = 1.11, 95% CI: 1.21–1.48). In the presence of children, cooking with wood, coal, or kerosene increased the risk of contracting rhinitis (OR = 1.31, 95% CI: 1.04–1.69), hay fever (OR = 1.21, 95% CI: 1.07–1.81). Heating homes using kerosene, wood, or coal leads to rhinitis ever, current rhinitis, and current rhinoconjuctiitis (OR = 0.65, 95% CI: 0.53–0.81) and hay fever (OR 0.65 95% CI: 0.53–0.81).
14.	Buthelezi et al. [34]	Cross-sectional	Men and women living in selected households in the study area.	245	Umlazi Township in the City of eThekwini, KwaZulu-Natal province, South Africa	Electric (electricity) and non-electric (wood, coal, gas, paraffin)	Upper Respiratory Tract Infections (URTI) and Lower Respiratory Tract Infections (LRTIs) Non-electric sources for heating (aOR = 3.6, 95% CI: 1.2–10.1, *p* < 0.05) and cooking (aOR = 2.9, 95% CI: 1.1–7.9, *p* < 0.05) was significantly associated with high prevalence of URTIs. Electric sources for heating was associated with prevalence of LRTIs (aOR = 2.7, 95% CI: 1.1–6.4, *p* < 0.05).
15.	Jafta et al. [51]	Case-control	Children aged 0–14 years diagnosed with pulmonary Tuberculosis (PTB) and without pulmonary Tuberculosis	234 children, 107 cases and 127 controls	Durban, South Africa	Dampness, secondhand smoke, PM_10_, NO_2_	Dampness (OR = 1.8, 95% CI: 1.01–3.1), cooking fuel type (OR = 2.6, 95% CI: 1.1–6.4), and SHS (OR = 1.7, 95% CI: 0.98–2.8), and PM_10_ (OR = 1.4, 95% CI: 0.8–2.3) were positively associated with PTB in children in the unadjusted analysis. In the adjusted analysis, visible dampness was significantly associated with PTB (aOR = 2.4, 95% CI: 1.1–5.0). However, the risk of PTB was lower for increase in NO_2_ concentration (aOR = 0.4; 95% CI: 0.2–0.8) and not significantly associated with increase in PM10 (aOR = 0.9; 95% CI: 0.5–1.8).
16.	Vanker et al. [52]	Longitudinal study	Pregnant women and infants	982 pregnant women and 986 infants	Mbekweni and Newman, South Africa	Particulate matter, carbon monoxide, nitrogen dioxide, volatile organic compounds.	Antenatal exposure to NO_2_ above ambient standards was associated with increased maternal nasopharyngeal carriage of *M. catarrhalis* when adjusted for clinical covariates as well as the other pollutants (aRR = 3.69, 95% CI = 1.27–10.73); Benzene exposure was associated with maternal *H. influenzae* carriage when adjusted for clinical covariates (aRR = 2.06, 95% CI: = 1.18–3.59) and tobacco smoke exposure almost doubled the risk of *S. pneumoniae* carriage in mothers (aRR = 1.73, 95% CI: 1.03–2.92); PM_10_ was associated with an increased risk of *H. influenzae* at 6 months, (aRR = 1.60, 95% CI: 1.04–2.46) and *M. catarrhalis* at 12 months (aRR = 1.39, 95% CI: 1.02–1.90), and NO_2_ with Gram-negative

## Data Availability

Not applicable.
